# Compatible and Incompatible Pollen-Styles Interaction in *Pyrus communis* L. Show Different Transglutaminase Features, Polyamine Pattern and Metabolomics Profiles

**DOI:** 10.3389/fpls.2019.00741

**Published:** 2019-06-07

**Authors:** Manuela Mandrone, Fabiana Antognoni, Iris Aloisi, Giulia Potente, Ferruccio Poli, Giampiero Cai, Claudia Faleri, Luigi Parrotta, Stefano Del Duca

**Affiliations:** ^1^Department of Pharmacy and Biotechnology, University of Bologna, Bologna, Italy; ^2^Department for Life Quality Studies, University of Bologna, Rimini, Italy; ^3^Department of Biological, Geological and Environmental Sciences, University of Bologna, Bologna, Italy; ^4^Department of Life Sciences, University of Siena, Siena, Italy

**Keywords:** self-incompatibility, Pyrus communis, transglutaminase, ^1^H NMR-metabolomics, polyamines

## Abstract

Pollen-stigma interaction is a highly selective process, which leads to compatible or incompatible pollination, in the latter case, affecting quantitative and qualitative aspects of productivity in species of agronomic interest. While the genes and the corresponding protein partners involved in this highly specific pollen-stigma recognition have been studied, providing important insights into pollen-stigma recognition in self-incompatible (SI), many other factors involved in the SI response are not understood yet. This work concerns the study of transglutaminase (TGase), polyamines (PAs) pattern and metabolomic profiles following the pollination of *Pyrus communis* L. pistils with compatible and SI pollen in order to deepen their possible involvement in the reproduction of plants. Immunolocalization, abundance and activity of TGase as well as the content of free, soluble-conjugated and insoluble-bound PAs have been investigated. ^1^H NMR-profiling coupled with multivariate data treatment (PCA and PLS-DA) allowed to compare, for the first time, the metabolic patterns of not-pollinated and pollinated styles. Results clearly indicate that during the SI response TGase activity increases, resulting in the accumulation of PAs conjugated to hydroxycinnamic acids and other small molecules. Metabolomic analysis showed a remarkable differences between pollinated and not-pollinated styles, where, except for glucose, all the other metabolites where less concentrated. Moreover, styles pollinated with compatible pollen showed the highest amount of sucrose than SI pollinated ones, which, in turn, contained highest amount of all the other metabolites, including aromatic compounds, such as flavonoids and a cynnamoil derivative.

## Introduction

In order to prevent self-fertilization, plants have evolved different strategies, from the temporally asynchronous development of male and female reproductive organs, to their specific localization within the flower, up to genetic-based strategies called “self-incompatibility” (SI) (Barrett, 2003; Ashman et al., 2004). The latter process prevents self-fertilization by the rejection of pollen of the same species. Based on whether the gametophytic or the sporophytic genome will determine pollen rejection, SI is classified into gametophytic SI (GSI) or sporophytic SI (SSI) (Fujii et al., 2016). Most of SI systems are controlled by a single gene locus, the so-called “S locus” which presents multiple S-alleles, i.e., the pistil-S and pollen-S genes; however, several other genes, proteins and external factors are involved in the process of pollen acceptance/rejection (Serrano et al., 2015; Qu et al., 2017).

In the last decades, both genetic and molecular factors involved in SI have been studied in Malinae, as GSI affects both quantitative and qualitative aspects of productivity in agricultural species of great economic interest (Del Duca et al., 2019). In the GSI of Malinae, the stylar *S* locus plays an important role, since it encodes for glycoproteins with ribonuclease (S-RNase) activities that enter inside the pollen tube. Once inside, they are degraded in case of compatible pollen, allowing pollen tubes to grow, while in SI pollen the S-RNases are kept active, causing the degradation of pollen RNA and the cell death of pollen tube, which generally reaches only the upper third part of the style (De Franceschi et al., 2012).

Several evidences highlight the involvement of transglutaminase (TGase) in the process of pollen rejection, not only in Malinae (Gentile et al., 2012; Aloisi et al., 2016a; Del Duca et al., 2019). TGases are ubiquitous enzymes that catalyze the post-translational modification of proteins, through the incorporation of primary amines or by protein cross-linking, resulting often in high molecular mass products (Griffin et al., 2002). In plants, TGases are distributed in different cell compartments where they exert a structural or conformational role (Del Duca and Serafini-Fracassini, 2005; Serafini-Fracassini and Del Duca, 2008; Serafini-Fracassini et al., 2009). The post-translational modification of proteins by polyamines (PAs) and the catalysis of isopeptide bonds are the main TGase reaction studied in plant (Del Duca et al., 2000; Aloisi et al., 2016b). Although TGase could also catalyze the deamidation of endoglutamine residues of protein substrates, up to now this reaction has been scarcely considered in plants. TGase is a crucial factor for the growth of pollen tubes as is involved in the cross-link of several substrates (mainly cytoskeleton proteins) and in the post-translational modification of proteins (Di Sandro et al., 2010; Aloisi et al., 2016a). TGase activity increases during the SI response (Del Duca et al., 2010; Gentile et al., 2012) and programmed cell death (PCD)-related processes, when the stimulation of TGase activity is mainly due to the increase of Ca^2+^ (Della Mea et al., 2007). Recently, also in Malineae, PCD phenomena have been shown to occur as the consequence of SI response (Wang et al., 2010; Li et al., 2018).

Polyamines are essential for cell growth, and their contents in cells are maintained by biosynthesis, degradation and transport; PAs transport systems have been identified in different organisms, from bacteria to plants (Antognoni et al., 1999; Fujita and Shinozaki, 2014). Despite the wide array of investigations on PA roles in plant cells, little information is available on their role during the apical growth of pollen within the styles. PAs biosynthesis increased during pollen tube emergence and elongation and they are released in the external space (Bagni et al., 1981) where they affected RNAse activity (Speranza et al., 1984). Together with the decrease in free PAs, changes in the levels of PAs bound to low- and high-mass molecules take place inside the pollen tube (Antognoni and Bagni, 2008). Moreover, PAs are directly involved in ROS regulation, whose concentration is essential for pollen tube growth (Wu et al., 2010; Aloisi et al., 2015).

Metabolomics is a novel inductive approach, relying on untargeted analysis protocols, whose results are handled by multivariate data treatment. This workflow proved to be successful in several research fields, from human diagnostics and epidemiology to plant science (Wolfender et al., 2015; Anđelković et al., 2017), where it resulted particularly helpful to face the challenges posed by the complexity of natural product chemistry (Pauli et al., 2012). For instance, metabolomics has been employed to facilitate the identification of medicinal plants active principles (Gođevac et al., 2018; Mandrone et al., 2018).

In this work, styles of *Pyrus communis* L. cv Abbé Fétel were pollinated with compatible Williams pollen (AxW) and with incompatible Abbé Fétel pollen (AxA) in order to induce SI response.

As many changes occur (i.e., in the cell wall) during the growth of pollen tube inside the style, it is interesting to check if this is complemented by variation in metabolomics profile that has been analyzed in not-pollinated (NP) styles and in styles pollinated with compatible and SI pollen, at 48 h after pollination. Besides, the metabolomic analyses, performed for the first time on SI systems, will add knowledge enlarging the picture of factors involved in the SI process and will help to clarify what are the main actors playing into the SI response in the complex molecular networks of pear SI system. For example, it is reasonable to hypothesize changes in cell wall as well as in phenolic compounds or hydroxycinnamoyl-derivatives that could take place because of the pollen-style interaction.

As the cytoskeleton is a target of SI response (Thomas et al., 2006), and TGase affects the organization of microfilaments and microtubules (Del Duca et al., 1997, 2009), TGase amount, activity, and localization have been checked. In particular, analysis were carried out at different time intervals, from the first hours until 48 h after pollination. Moreover, given the role of PAs in fertilization process (Aloisi et al., 2016a), and the role of TGase as a mediator of PAs action (Del Duca et al., 2014a), their profiles and their changes in styles pollinated with compatible and SI pollen have been investigated.

## Materials and Methods

### Materials

All reagents were purchased from Sigma-Aldrich (Milan, Italy), except the deuterated solvents, which were purchased by Eurisotop (Cambridge, United Kingdom).

### Plant Material, *in vitro* and *in vivo* Pollen Germination and Sampling

Mature pollen of pear (*Pyrus communis* L. cv. “Abbé Fétel” and “Williams” was collected from plants grown in experimental plots at the University of Bologna (Dipartimento di Scienze e Tecnologie Agro-Alimentari, University of Bologna). Handling, storage, pollen hydration and 2 h germination were performed as previously reported (Aloisi et al., 2015, 2017). Styles were collected from flowers of *Pyrus communis* “Abbé Fétel” at the balloon stage. For pollination, about 15 sprigs, each with at least 50 flowers at the balloon stage, were collected from a maximum of 20 different Abate trees. These were grown in different positions in the orchard and flowers were collected from various positions on the same tree. Sprigs with flowers were placed in a beaker with water, and anthers were removed from each flower in the laboratory. Once emasculated, they were pollinated with compatible and incompatible pollen. In the compatible crosses, styles of Abbé Fétel (S-genotype: S104/S105) were pollinated with William (S101/S102) pollen (AxW); in the incompatible ones, styles of Abbé Fétel were self-pollinated (AxA). After 48 h, pollinated pistils were collected and immediately frozen in liquid nitrogen, then transferred at -80°C. Equal numbers of pistils were randomly chosen to create pooled samples of 20 pistils. The procedure was performed four times to generate four biological replicates for the analysis of metabolomics, total flavonoids, PAs and TGase activity. Three technical replicates were performed.

### Staining of Callose With Aniline Blue

Styles of pear were immediately fixed in 3% glutaraldehyde in 44 mM cacodylate buffer, pH 7.2, for 2 h at room temperature. Samples were washed rapidly with the cacodylate buffer and placed in a solution of 8 N NaOH for 2 h at room temperature. Samples were then washed in distilled water for 10 min and stained with 0.1% aniline blue in 0.1 M KH_2_PO_4_ for 2 h; then, samples were squashed by placing them between a glass slide and a coverslip and by applying a gentle pressure. Observations were made with a 340 nm UV filter-equipped fluorescence microscope (Zeiss Axiovision).

### Transglutaminase Quantification and Enzyme Activity Assay

Pollen and pistils proteins were extracted according to literature (Gentile et al., 2012). Briefly, proteins were solubilised at 4°C in extraction buffer (10 mg ml^-1^) containing 100 mM Tris–HCl pH 8.5, 2 mM dithiothreitol (DTT), 0.5 mM ethylenediaminetetraacetic acid (EDTA) and 0.2 mM phenylmethylsulphonylfluoride (PMSF) in a Potter–Elvehjem homogenizer. Large cell debris were removed from the total homogenate by centrifugation at 10,000 *g* for 10 min at 4°C. Protein concentration was estimated on the supernatant by the Bradford (1976) method with bovine serum albumin (BSA) as the standard protein.

ELISA assay was carried out in triplicate as described previously (Paris et al., 2017). Briefly, a 96-wells plate was incubated overnight at 4°C with extracted proteins (50 μl/well). Wells were washed twice with PBS (Phosphate buffered saline) buffer then incubated 1 h at RT with 200 μl/well of 5% defatted milk dissolved in PBS. Wells were washed twice and the mouse monoclonal antibody ID10 (Nottingham Trent University) was added after dilution (1:500) in PBS for 2 h at RT. Wells were washed with 0.05% Tween 80 in PBS three times and incubated with the secondary peroxidase conjugated antibody (1:500) for 1 h at RT. After washing, the substrate solution of 0.3 mM 3,3′,5,5-Tetramethylbenzidine (TMB) (10 mg/mL of dimethyl sulfoxide) and 0.03% (v/v) hydrogen peroxide (H_2_O_2_) in 100 mM sodium acetate (CH_3_COONa) pH 6.0 was added. The staining development was stopped after maximum 30 min with 50 μl per well of 5 N sulfuric acid (H_2_SO_4_). The absorbance was read at 450 nm using a Wallac Victor Multiscan ELISA (Perkin Elmer).

The *in vitro* TGase activity was measured on extracts of ungerminated pollen (UGP) and germinated (GP) *in vitro* for 2 h as well as on NP (not pollinated) and pollinated styles after 6, 24 and 48 h from pollination, by the conjugation of biotinylated cadaverine to exogenous substrates *N, N*′-dimethylcasein or fibronectin as previously described (Di Sandro et al., 2010; Del Duca et al., 2018). Specific activity was determined as a change in A_450_ of 0.1 per hour per mg of pollen after subtraction of the value of the controls treated with 20mM EGTA [ethylene glycol-bis(β-aminoethyl ether)-N,N,N′,N′-tetraacetic acid].

### Immuno-Localisation of TGase

Compatible (AxW) and incompatible (AxA) styles of pear were pollinated and after 24 and 48 h frozen at -80°C. Samples were directly thawed in a buffer solution (100 mM Pipes pH 6.8, 10 mM EGTA, 10 mM MgCl_2_, 0.1% NaN_3_) plus detergent and fixative (0.05% Triton X-100, 1.5% paraformaldehyde, 0.05% glutaraldehyde) for 30 min on ice and then at 4°C for additional 30 min. For localization of TGase, fixed pear styles were cut along their length and placed in the buffer solution containing 0.75% cellulysin and 0.75% pectinase for 7 min. For immunofluorescence microscopy, samples were washed in the above buffer and incubated with the anti-TGase antibody ID10 (Nottingham Trent University) diluted 1:20 in the buffer; incubation was 1 h at 37°C. This antibody has been shown to immunoreact with partially purified TGase extracted from apple pollen (Del Duca et al., 2009; Di Sandro et al., 2010). After washing with the buffer, styles were incubated with the secondary antibody goat anti-mouse FITC-conjugated, diluted 1:50 in the buffer solution, for 45 min at 37°C in the dark. Samples were observed with a Zeiss Axiophot fluorescence microscope equipped with a MRm video camera and a 63× oil-immersion objective. For electron microscopy, samples were embedded in resin and sectioned as described (Parrotta et al., 2018). In this case, the ID10 antibody was used at 1:5 dilution while the goat anti-mouse 10 nm gold-conjugated secondary antibody was used at 1:100. Samples were observed with a Philips Morgagni 268D electron microscope equipped with a MegaView II video camera.

### HPLC Analysis of Polyamines

HPLC PAs analysis was performed to investigate the content of the diamines Putrescine (Put) and Cadaverine (Cad), the triamine Spermidine (Spd) and the tetramine Spermine (Spm). Dried styles (0.025 g) were extracted in 100 vol. of 4% (w/v) cold perchloric acid (PCA), left on ice for 1 h and centrifuged at 15.000 × *g* for 30 min at 4°C. Supernatant was measured and used for analysis of Free (F) and soluble-conjugated (SC) PAs. After washing the pellets twice with cold PCA, it was resuspended in the original volume, and used for analysis of insoluble-bound (IB) PAs.

For SC and IB fractions, replicates (0.3 mL) of supernatant and pellet suspension, respectively, were subjected to hydrolysis with 6N HCl for 24 h at RT, in order to free polyamines from their conjugates, as described previously (Torrigiani et al., 1989). After hydrolysis, samples were brought to dryness at 140°C and 0.3 mL of 4% PCA were added.

Aliquots (0.2 mL) of supernatant, hydrolysed supernatant and hydrolysed resuspended pellet were dansylated according to Smith and Davies (1985) with minor modifications. 0.2 mL of dansylchloride (5 mg/mL in acetone) and 0.2 mL of a saturated solution of Na_2_CO_3_ were added to samples and incubated at 60°C for 1 h in the dark. Then, after addition of 0.1 mL proline (15 mg/mL), samples were incubated for 30 min at RT in the dark. Dansylpolyamines were then extracted in toluene, the solvent was evaporated and samples were resuspended in 0.2 mL acetonitrile. Standard polyamines and heptamethylendiamine, as an internal standard (all by SIGMA-Aldrich) were subjected to the same procedure.

HPLC analysis was carried out on a Jasco system (Jasco–Tokyo, Japan) consisting of PU-4180 pump, FP-821 detector, and an AS-4050 autosampler. Stationary phase was an Agilent (Santa Clara, CA, United States) Zorbax Eclipse Plus C18 reversed-phase column (100 mm × 3 mm I.D., 3.5 μm), and mobile phase was a mixture of acetonitrile and water. Elution was carried out with a step gradient as follows: 60 to 70% acetonitrile in 5.5 min, 70 to 80% acetonitrile in 1.5 min, 80 to 100% acetonitrile in 2 min, 100 to 100% acetonitrile in 2 min, 100 to 70% in 2 min, and 70 to 60% in 2 min at a flow rate of 1.5 mL/min. Eluted peaks were detected by the FP fluorimeter at 365 nm excitation, and 510 nm emission, and data signals were acquired and processed through the software Chromnav 2.0 (Jasco). Results were expressed as nmol/g DW.

### Extraction for Metabolomic Analysis and NMR Measurements

Thirty milligrams of freeze-dried styles were extracted using 1 mL of a blend (1:1) of phosphate buffer (90 mM, pH 6.0) in D_2_O containing 0.01% trimethylsilylpropionic-2,2,3,3-d*_*4*_* acid sodium salt (TMSP) and CD_3_OD by ultrasonication for 25 min (Verpoorte et al., 2007; Kim et al., 2010). After this procedure, samples were centrifuged for 10 min (1700 × *g*) and 700 μL of the supernatant were transferred into NMR tubes for the analysis. The ^1^H NMR spectra were recorded at 25 °C on a Varian Inova 600 MHz NMR instrument (600 MHz operating at the ^1^H frequency) equipped with an indirect triple resonance probe, CD_3_OD was used for internal lock. Relaxation delay was 2.0 s, observed pulse 5.80 μs, number of scans 256, acquisition time 16 min, and spectral width of 9595.78 Hz (corresponding to δ 16.0). A presaturation sequence (PRESAT) was used to suppress the residual H_2_O signal at δ 4.83 (power = -6dB, presaturation delay 2 s).

### NMR Processing and Multivariate Data Treatment

Free Induction Decays (FIDs) were Fourier transformed, and the resulting spectra were phased, baseline-corrected and calibrated to TMSP at δ 0.0, spectral intensities were reduced to integrated regions of equal width (δ 0.04) corresponding to the region from δ 0.0 to 10.0, with scaling on standard at δ 0.0 using the ^1^H NMR Mestrenova software (Mestrelab Research, Spain). The metabolites were identified on the bases of an in-house library and comparison with literature (Verpoorte et al., 2007; Scognamiglio et al., 2015; Mandrone et al., 2017).

The regions of δ 5–4.5 and 3.34–3.30 were excluded from the analysis because of the residual solvents signals. For multivariate analysis (PCA, PLS-DA), data were subjected to Pareto scaling. The models were developed using SIMCA-P software (v. 15.0, Umetrics, Sweden).

### Total Flavonoids Assay

The assays was performed in Spectrophotometer Jasco V-530 as described by Chiocchio et al. (2018) with slight modifications. Thirty milligrams of freeze-dried sample were extracted using 1 mL of a MeOH/H_2_O (50:50) by ultrasonication for 25 min. After this procedure, samples were centrifuged for 10 min (1700 × *g*) and 50 μL of the supernatant were transferred were mixed with 450 μL of MeOH and 500 μL of AlCl_3_ (2% w/v in methanol). Rutin stock solutions (from 1 to 100 μg/mL) were prepared in MeOH (from 1 mg/mL rutin solution in DMSO) and 50 μL of each stock were treated as above described for the samples, in order to obtain a calibration curve. The absorption at 430 nm was recorded after incubation (15 min) at room temperature. Total flavonoid content of the extracts was calculated by interpolation in the calibration curve, and expressed in terms of mg RE (rutin equivalent)/g of samples (dried weight of plant material).

### Statistical Analysis

Data from polyamine, total flavonoids analysis and TGase activity were statistically analyzed by the one-way Analysis of Variance (ANOVA), followed by Tukey’s Multiple Comparison Test, using Graph Pad Prism package (v. 5.01 for Windows; GraphPad Software, San Diego, CA, United States). Metabolomics data were subjected to Pareto scaling and the models (PCA, PLS-DA) were developed using SIMCA-P software (v. 15.0, Umetrics, Sweden).

## Results

### Callose Staining With Aniline Blue in Styles and Pollinated Styles

A preliminary analysis was carried out to check the timing of SI response, thus styles of pear (NP and pollinated with compatible and incompatible pollen) were stained with aniline blue for callose reaction 48 h after pollination. Compatible pollinated styles (AxW) showed several pollen grains (PG) while adhering on the stigmatic papillae (P). Most of pollen grains showed a pollen tube (arrows) that penetrate along the style of flowers grown on entire plants (Supplementary Figures 1A–C). The self-fertilized styles (AxA) showed several pollen grains (PG) deposited on the surface of stigmatic papillae (P). Unlike the compatible cross, only a few pollen grains emitted a pollen tube (arrows) that were hardly observable along the style; in addition, the few pollen tubes appeared as bent and distorted (Supplementary Figures 2A–C). In NP styles, pollen grains as well as pollen tubes were not visible (Supplementary Figures 3A,B). Definitely, no differences in timing of SI response have been observed when pollinated styles belong to sprigs (Supplementary Figures 4A–C) or to entire planta (Supplementary Figures 1–3).

### Transglutaminase Activity

**FIGURE 1:**
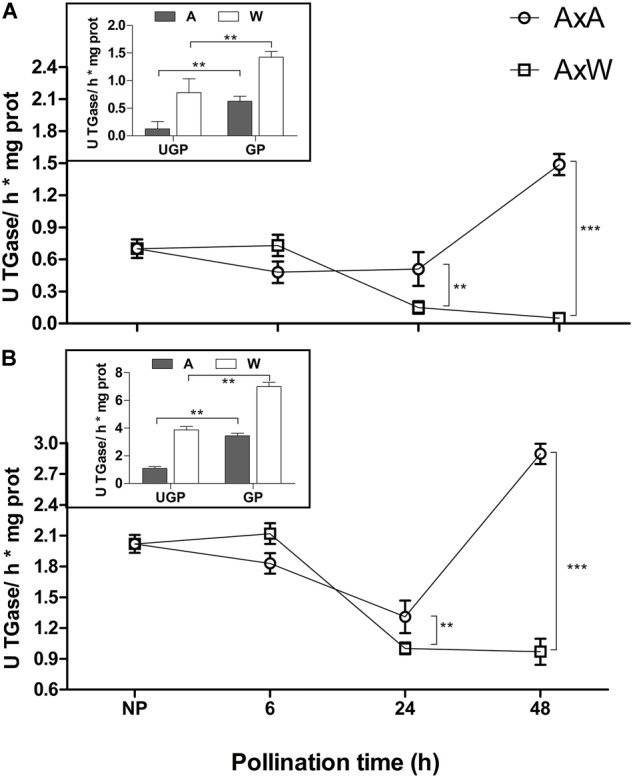
Transglutaminase activity was tested in NP, AxA and AxW styles of *Pyrus communis* on both N, *N*′-dimethylcasein **(A)** and fibronectin **(B)**. Hydrated and *in vitro* germinated pollen were also analyzed for TGase activity (inserts). TGase activity is expressed as units (U) of specific activity (means ± SD) per mg of protein. Means ± SD of three experiments analyzed in triplicate are reported. Samples indicated with asterisks were significantly different each other. The data were analyzed by one-way ANOVA and Tukey’s post-test. A *p* value < 0.05 was regarded as statistically significant. ^∗∗^*p* < 0.01. ^∗∗∗^*p* < 0.001.

Transglutaminase activity was measured in NP and pollinated styles using both *N, N*′-dimethylcasein and fibronectin, two well-known TGase substrates from mammal sources (Figure 1A,B). Since it is not possible to distinguish if the measured activity derives from the enzyme present in the germinated pollen or in the styles, also hydrated and *in vitro* germinated pollens were analyzed for TGase activity (Figure 1A,B, inserts). No differences were observed 6 h after pollination neither in AxA, nor in AxW pollinated styles. After 24 h, both types of pollinated styles showed a significant decrease in TGase activity compared with NP styles, with a more pronounced effect in AxW than AxA. After 48 h, TGase activity in SI model was much higher than in the compatible one, reaching a ten-fold difference when dimethylcasein was used as a substrate (Figure 1A). In case of fibronectin (Figure 1B), the activity was higher when compared to *N, N*′-dimethylcasein for all samples, allowing to hypothesize that the former is a better substrate for pollen TGase than the latter. In both cases, AxA showed much higher (5- to 10-fold) TGase activity compared to AxW.

**FIGURE 2:**
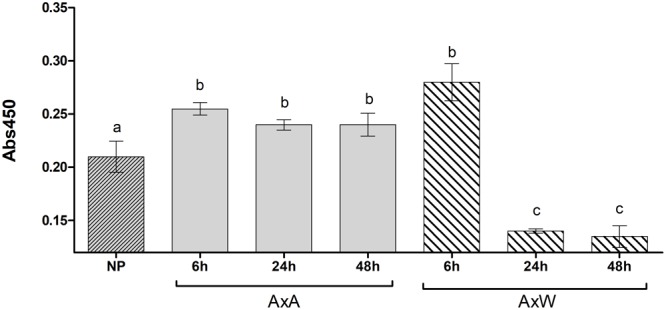
Transglutaminase relative quantification, expressed as absorbance units (Abs 450 nm) in NP, AxA and AxW styles of *Pyrus communis*. Means ± SD of three experiments analyzed in triplicate are reported. Samples indicated with different letters were significantly different each other. The data were analyzed by one-way ANOVA and Tukey’s post-test. A *p* value < 0.05 was regarded as statistically significant. Bars marked with the same letter are not significantly different.

Transglutaminase activity was partially mirrored by the enzyme amount. In fact, the amount of TGase present during the pollination phases from 0 to 48 h was evaluated with an ELISA test (Figure 2) by ID10. Results showed that, at all-time points (6, 24, and 48 h), AxA showed a significantly higher TGase amount compared to NP styles. Conversely, in AxW, only after 6 h a significantly higher amount compared to control was observed, whereas at 24 and 48 h the TGase amount definitely decreased compared to both control and AxA (Figure 2). The greater presence of TGase observed at 6 h both in AxA and AxW in respect to control did not translate into a higher value of enzymatic activity. On the contrary, at 48 h the amount of TGase both in AxA and AxW mirrored the level of its activity, showing the highest level of TGase activity in AxA and the lowest in AxW (Figure 1A,B). These data showed that SI stimulated TGase activity. Finally, TGase present in pollen was stimulated by germination, being this enzyme activity higher in GP than UGP. This activity was higher in William in respect to Abate pollen, probably due to different germination rates (Figure 1A,B, inserts).

### Immuno-Localization of TGase

**FIGURE 3:**
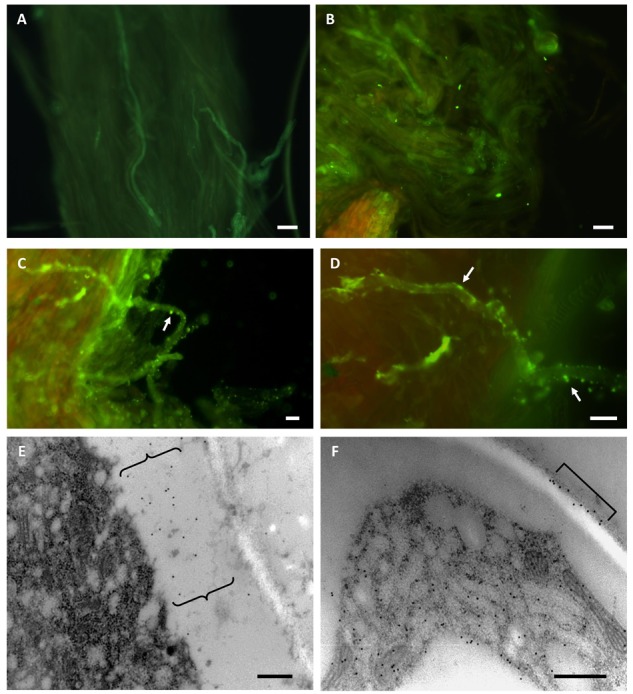
Compatible **(A,B)** and incompatible **(C–F)** pollinated styles of pear analyzed with ID10 anti-TGase antibody. **A,B:** examples of AxW pollinated styles labeled with ID10 in indirect immunofluorescence. In addition to a diffuse background, no specific signal was detected. **C,D:** examples of AxA pollinated pear styles investigated in indirect immunofluorescence with ID10 antibody. Pollen tubes have a punctiform labeling along their surface (arrows). Bars in **A–D**: 10 μm. **E,F**: Immunogold labeling with ID10 antibody on AxA pollinated styles; the images show details of pollen tubes with cell wall-associated labeling in the form of distinct clusters (curly brackets in **E**, square bracket in **F**). Bars in **E,F**: 500 nm.

The presence of TGase was investigated in pear styles pollinated in both a compatible and incompatible way (Figure 3). In the case of compatible styles (Figure 3A,B), immunofluorescence investigations with the ID10 antibody did not produce specific signals, apart from some backgrounds. Conversely, incompatible styles (Figure 3C,D), when analyzed in indirect immunofluorescence with ID10 antibody, showed pollen tubes with an intense and specific labeling along their entire surface (arrows). The labeling had a predominantly punctiform appearance and was associated with the peripheral region of pollen tubes, i.e., the plasma membrane and/or cell wall. To get more details on the distribution of TGase, analyses were carried out using the immunogold labeling technique (Figure 3E,F). In compatible pollinated styles, no evidence of signals or specific signals occurred (data not shown). On the contrary, in incompatible pollinated styles, labeling with ID10 antibody showed evidence of signal associated with the cell wall of pollen tubes. The signal was distributed in the form of distinct clusters present in the thickness of the cell wall (Figure 3E) or on the external side (Figure 3F).

### Polyamine Levels

**FIGURE 4:**
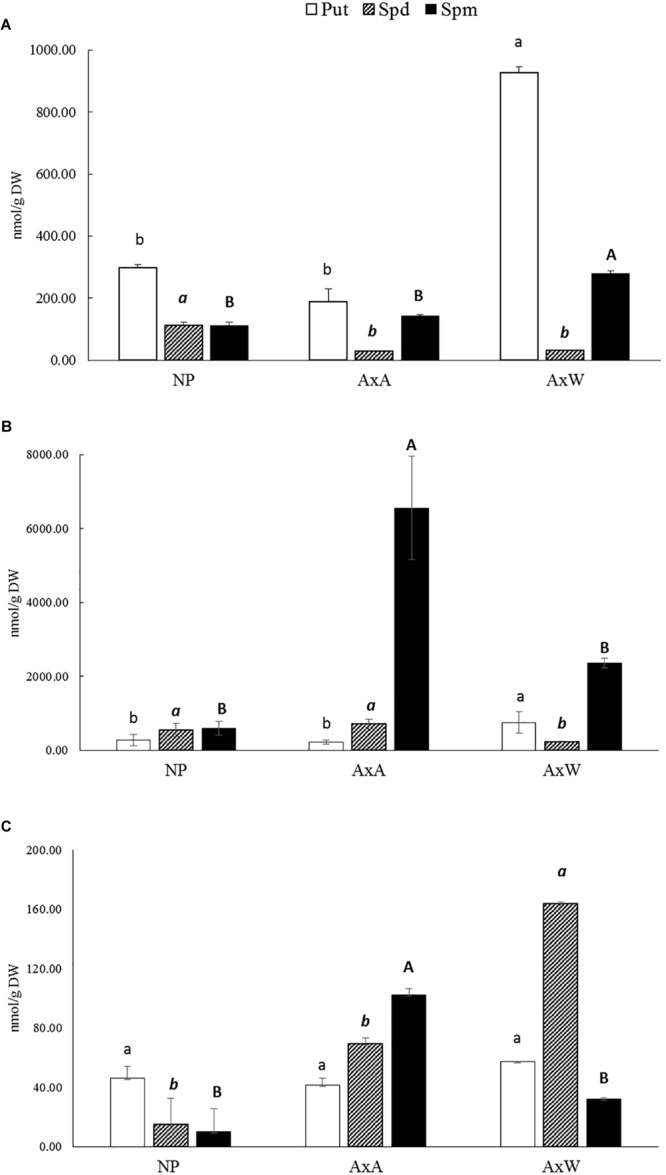
Free **(A)**, soluble-conjugated **(B)** and insoluble-bound **(C)** polyamine pattern in NP, AxA, and AxW styles of *Pyrus communis* cv Abbé Fétel. For each polyamine, different letters (lowercase, italic, and bold) indicate significant differences at *P* < 0.05. Means ± SD of three experiments analyzed in triplicate are reported.

The pattern of PAs in the F, SC, and IB forms was investigated in NP and pollinated styles AxA and AxW, respectively (Figure 4A–C). Put, Spd, and Spm were the only polyamines detected, while Cad was not found, neither in the former, nor in the latter types of styles (data not shown).

As concerns the F fraction, Put was the most abundant polyamine in all samples; AxW showed the highest levels, reaching values about 4.9 and 3.1 higher than AxA and NP styles, respectively (Figure 4A). As regards Spd and Spm, they were present at similar levels in NP styles, whereas in pollinated ones Spm was higher than Spd, with a ratio of 9.1 and 4.8 in AxW and AxA, respectively (Figure 4A).

Spermine was the major polyamine present in the SC fraction, i.e., conjugated through covalent bonding to low molecular weight compounds, followed by Spd and Put (Figure 4B). Spm levels turned much higher in pollinated compared to NP styles; in particular, SI styles (AxA) had a 2.7 times higher SC Spm than AxW. These styles also had a higher SC Spd level compared to AxW styles, while rather similar compared to NP ones. Thus, the predominant form of Spd and Spm in pollinated styles was the soluble-conjugated one, which turned much higher, compared to the free form (Figure 4B). In particular, in AxA styles, soluble-conjugated Spm was more than 40 times higher than the free form. Conversely, Put was present at rather similar levels in the F and SC form in all types of styles.

As concerns the IB fraction, this represent, generally speaking, the less abundant form of PAs in all styles (Figure 4C). Put in the bound form was much lower than the free and SC ones, with very similar levels in NP, AxA and AxW styles. Conversely, much pronounced differences were observed in bound Spd and Spm, both being more concentrated in pollinated than in NP styles. Spd bound to high molecular mass compounds was more than 2-fold higher in AxW compared to AxA, while for bound Spm an opposite trend was observed, with higher levels in the latter than in the former (Figure 4C).

### ^1^H NMR-Based Metabolomic Analysis

The ^1^H NMR-based metabolome of four samples for each class (NP, AxA and AxW) were analyzed and compared. Each sample was constituted of pooled material from twenty different flowers.

**FIGURE 5:**
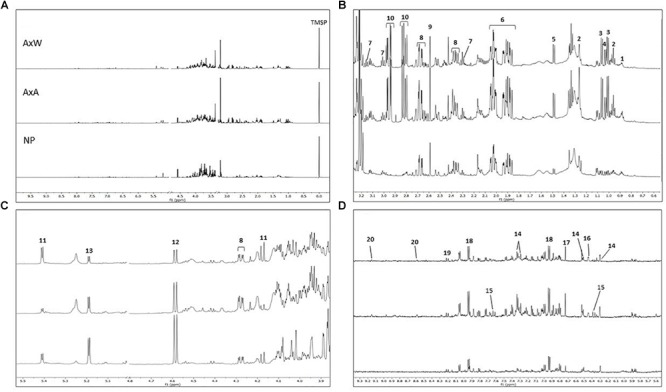
**(A)** From top to bottom: AxW; AxA and NP ^1^H NMR full spectra of representative samples belonging to the three different classes (residual solvent signals have been removed; TMSP = standard). Extended spectral regions **(B)** form δ 0.5 to 3.3; **(C)** form δ 3.9 to 5.5; **(D)** δ 5.6 to 9.3. Numbers indicated diagnostic signals of the most variated metabolites: 1, fatty acids; 2, α-linolenic acids; 3, valine; 4, isoleucine; 5, alanine; 6, quinic acid; 7, GABA; 8, malic acid; 9, succininc acid;10, glutamine; 11, β-glucose; 12, sucrose; 13, α-glucose; 14, quercetin like flavonoids; 15, cynnamoyl derivative; 16, shikimic acid; 17, fumaric acid; 18, p-hydroxybenzoyc acid; 19, kaempherol like flavonoid; 20, trigonelline.

**FIGURE 6:**
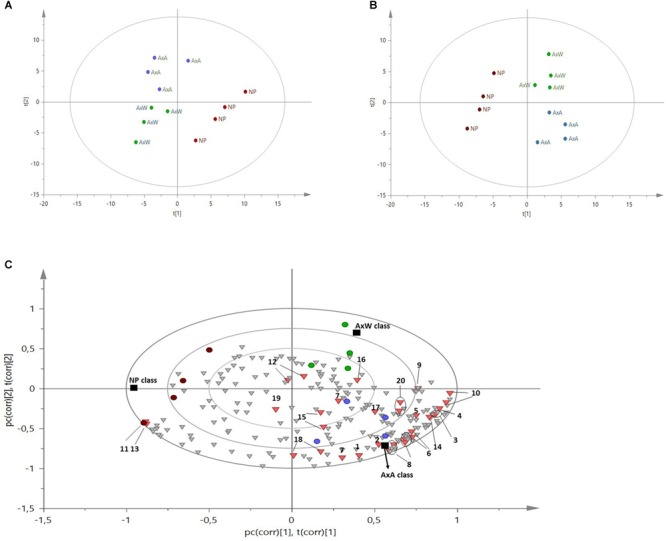
**(A)** 1H NMR based-PCA Score Scatter Plot, colored according to the three categories of samples. Each category included four replicates. Although the model is unsupervised, it individuated three different metabolic patterns among the given samples. **(B)** PLS-DA Score Scatter Plot, supervised model where the three classes (NP, AxW and AxA) were given as y variables. **(C)** B-Plot of the PLS-DA model, showing which NMR signals determined the grouping of the three classes indicated by the Score Scatter Plot. Black squares represent the three classes, dots are the 12 analyzed samples, inverted triangles are binned NMR signals and, among them, the red ones are the diagnostic signals of the most important metabolites. A general increase of all metabolites is observed in AxA, followed by AxW, while NP is less enriched in all metabolome, except glucose, which signals increase along component t[1]. Pollinated samples variate along the component t[2], indicating that AxW is more enriched in sucrose than AxA (this metabolite increases on positive component t[2]). 1, fatty acids; 2, α-linolenic acids; 3, valine; 4, isoleucine; 5, alanine; 6, quinic acid; 7, GABA; 8, malic acid; 9, succininc acid;10, glutamine; 11, β-glucose; 12, sucrose; 13, α-glucose; 14, quercetin like flavonoids; 15, cynnamoyl derivative; 16, shikimic acid; 17, fumaric acid; 18, p-hydroxybenzoyc acid; 19, kaempherol like flavonoid; 20, trigonelline.

The ^1^H NMR spectra were registered and processed in order to be suitable for multivariate data analysis. A representative spectrum for each class of samples is showed in Figure 5 and diagnostic signals of all metabolites are reported in Table 1. Firstly, PCA (an unsupervised model) was built with observation (*N*) of 12 and 212 variables (*x*) constituted by bucketed NMR signals, obtaining a R2X(cum) of 0.942 and a Q2X(cum) of 0.782. As showed by the PCA Score Scatter Plot (Figure 6A), a clear grouping of the three classes was identified (Figure 6A), indicating the occurrence of metabolomic differences among them.

**Table 1:** ^1^H NMR spectral references of the metabolites mentioned in this work, and resulting, according to multivariate data analysis, highly variated among the three classes NP, AxW and AxA of *Pyrus communis* samples.

Metabolites	Chemical shifts (δ); number and position of protons; multiplicity; coupling constants (Hz)
Alanine	1.48 (3H-β, d, J = 7.2 Hz)
Caffeoyil-moiety	7.62 (1H-7, d, J = 15.9 Hz), 6.38 (1H-8, d, J = 15.9 Hz)
Fatty acids	0.88 (H-x, t, J = 7.5 Hz)
Fumaric acid^a^	6.7 (1H-2 and 1H-3; s)
GABA	3.12 (2H-4, t, J = 7.5 Hz), 2.30 (2H-2, t, J = 7.5 Hz), 1.92 (2H-3 m)
α-glucose	5.20 (1H-1, d, J = 3.8 Hz)
β-glucose	4.59 (1H-1, d, J = 7.9 Hz)
Glutamine	2.82 (2H-γ, m); 2.95 (2H-β, m)
p-hydroxy benzoic acid	7.94 (1H-3, 1H-5, d, J = 8.8 Hz), 6.94 (1H-2, 1H-6, d, J = 8.8 Hz)
Isoleucine	1.06 (3H-β′, d, J = 6.8 Hz)
Kaempferol-moiety	8.04 (1H-2′ and 1H-6′, d, J = 8.6 Hz)
α-linolenic acids	0.96 (H-x, t, J = 7.5 Hz), 1.30 (CH_2_ brs)
Malic acid^a^	4.28 (1H-2, dd, J = 6.6, 4.7 Hz), 2.68 (1H3-a, dd, J = 16.6, 4.7 Hz), 2.36 (1H3-b, dd, J = 16.6, 6.6 Hz)
Quinic acid	1.87 (1H-2a, m); 1.96 (1H-6a, m); 2.01 (1H-2-b, m); 2.02 (1H-6-b, m), 3.40 (1H-4, ov); 4.00 (1H-3, ov); 4.11 (1H-5, ov)
Shikimic acid	2.19 (1H-6a, dd, J = 18.0, 5.1); 2.74 (1H-6b, dd, J = 18.0, 5.1); 3.99 (1H-5, m); 4.40 (1H-3, dd, J = 4.2, 4.2); 6.45 (1H-2, m)
Succinic acid^a^	2.43 (2H-2 and 2H-3, s)
Sucrose	5.40 (1H-1, d, J = 3.8 Hz), 4.17 (1H-3′, d, J = 8.5 Hz)
Trigonelline	9.14 (1H-2, s); 8.87 (1H-5, m)
Valine	1.00 (3H-β′, d, J = 6.8 Hz), 1.07 (3H-β″, d, J = 6.8 Hz)

*^*a*^Changeable by pH and concentration.*

To deeply and easily analyze these differences, a supervised model was built (PLS-DA) with *N* = 12 and 215 variables (*x* = 212 and *y* = 3, namely the three above mentioned classes). The first five components explained the maximum of variance and the model yield a R2X(cum) of 0.952, R2Y(cum) of 0.979 and a Q2(cum) of 0.873. The model was further validated by permutation test (using 100 permutations), which gives R2Y(cum) of 0.98 and Q2(cum) of 0.84, while the intercepts were 0.64 and -0.42 for R2 and Q2, respectively.

Samples belonging to the NP class were strongly distanced from the other two classes, being clustered along the negative component t[1] (Figure 6B,C). This indicated the globally less enriched spectrum (lowest amount of both primary and secondary metabolites) of this class in comparison to pollinated classes (AxA and AxW). The only exception was constituted by signals related to glucose, which increase in NP samples. More specifically, NP showed a lowest content of aliphatic amino acids (alanine, valine, isoleucine), glutamine, fatty acids, α-linoleic-acid, organic acid (quinic acid, GABA, succinic acid, shikimic acid, malic acid, *p*-hydroxybenzoyc acid), sucrose, trigonelline alkaloid and aromatic spectral signals probably due to cynnamoil derivatives and flavonoids, such as quercetin and kaempferol derivatives.

**FIGURE 7:**
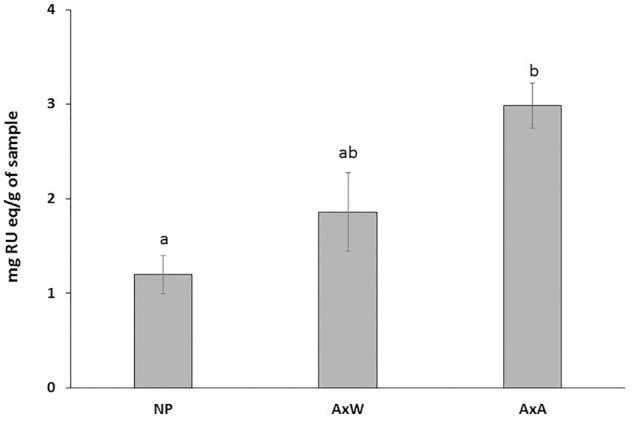
Total flavonoid content of the three classes. Data are expressed in RU eq (rutin equivalent)/g of dry plant material. AxA samples showed a significant increase in total flavonoid content. Means ± SD of three experiments analyzed in triplicate are reported. The data were analyzed by one-way ANOVA and Tukey’s post-test. A *p* value < 0.05 was regarded as statistically significant as indicated by the different letters a, b.

Moreover, samples belonging to AxA and AxW classes were distanced along the component t[2], indicating that class AxW was more enriched in sucrose than AxA, while the latter showed highest concentration of all the other metabolites, including aromatic signals ascribable to cynnamoil derivatives and flavonoids. To further confirm this latter data, total flavonoid content of the three classes was also analyzed (Figure 7) showing, in fact, a significant increasing of these metabolites in AxA samples, which differ by (*P* < 0.05) from NP.

## Discussion

Cytosolic and cell wall-associated TGases activity during pollen tube growth have been reported (Del Duca et al., 2014b), suggesting its potential role in the modification of both the cytoskeleton (Del Duca et al., 2010) and components of the cell wall matrix, such as proteins, polysaccharides or glycoproteins (Waffenschmidt et al., 1999). Moreover, previous evidences have also demonstrated an alteration in TGase activity occurring during the SI reaction (Del Duca et al., 2010).

In this work, the staining of NP and pollinated styles with aniline blue confirmed the timing of the SI response according to literature data (Del Duca et al., 2010). In order to extend knowledge on TGases activity during SI, the distribution of this enzyme and its activity were monitored during 48 h. The obtained results highlighted changes in TGase localization after SI induction, generating aggregates in the cell wall, visible with confocal analysis. These aggregates were not present in compatible pollinated styles. These data might suggest the involvement of TGase in cell wall stiffening, leading to the arrest of pollen tube growth.

A key event following stresses or insults, including SI induction is the increase of Ca^2+^ levels up to mM concentrations (Griffin et al., 2002; Ranty et al., 2016). Being TGase a Ca^2+^-dependent enzyme, this ion is likely involved in up-regulation of TGase activity, whose catalysis takes place from 20 nM Ca^2+^ onward, leading, when the concentration increase up to mM, to a massive protein cross-linking, and consequent formation of protein aggregates (Griffin et al., 2002). The increased activity of TGase strongly affects the structure of cytosolic proteins such as actin and tubulin (Del Duca et al., 2009), resulting in cytoskeleton rearrangements, already reported in *Pyrus* and other SI models (Staiger and Franklin-Tong, 2003; Del Duca et al., 2009). These TGase-mediated rearrangements of cytosolic proteins are necessary for the proper growth of the pollen tube, and an upregulation of TGase could play a crucial role in SI response.

In addition to protein cross-linking, TGase activity modifies proteins conformation and surface charge also by PAs-protein conjugation. PAs are found both as free amines in cell compartments and conjugated to low molecular weight metabolites (e.g., hydroxycinnamoyl-derivatives), which are involved in pollen cell wall organization (Yang et al., 2007; Grienenberger et al., 2009) and fertilization (Elejalde-Palmett et al., 2015). Among these PAs-conjugated metabolites, hydroxycinnamoyl-amides (HCAs) are of great importance, since they modulate the strength of pollen cell wall, creating a bridge among cell wall molecules, especially lignins and hemicelluloses (Lam et al., 1992). Moreover, HCAs reduce the cytotoxicity of free hydroxycinnamic acids, due to their lipophilic character, known to inhibit growth (Kefeli et al., 2003).

Together with other metabolites, such as flavonol glycosides, HCAs are highly conserved in Angiosperm, conferring pollen coat structural characteristics (Fellenberg and Vogt, 2015). The amine moiety of HCAs derives from aromatic monoamines (such as tyramine, tryptamine) or aliphatic PAs (such as Put, Spd, Spm). In *M. domestica* HCAs are present in pollen coat (Elejalde-Palmett et al., 2015), and exhert also a protective role toward biotic and abiotic stressors.

Since the highest activity of TGase was detected at 48 h, at this time point PAs content was analyzed, showing a different pattern of PAs between AxA and AxW, with a higher level of free Put and Spm in compatible pollination compared to SI.

In *Pyrus communis*, previous results showed that PAs content changes during *in vitro* pollen germination, depending on both type of PA and their form (F, SC, IB) (Del Duca et al., 2010). According to these data, Put, Spd, and Spm were the only PAs detected in styles, as confirmed by recent bibliography about PAs in the early stages of fruit development (Quinet et al., 2019). This suggests a possible role for these compounds in the pollen-style interaction during the fertilization process (Aloisi et al., 2016a). These data are in agreement with the protective effect of PAs toward RNA, exerting an inhibitory role on the of S-RNase activity (Speranza et al., 1984; Li et al., 2018), which has been reported in different plant models (Altman, 1982; MccCre and Franklin-Tong, 2006; Wang et al., 2010; Li et al., 2018). PAs are also regulators of cytosolic Ca^2+^ (Alcázar et al., 2010), leading to the above- mentioned rearrangements of the actin cytoskeleton mediated by TGase (Aloisi et al., 2017).

Contrary to free PAs pattern, AxA showed a three-fold increase in SC-Spm compared to AxW, allowing hypothesizing its involvement in pollen tube growth inhibition. In fact, SC-PAs represent the product of PAs linkage to HCAs or to amino acids and/or peptides with MW < 5 kDa. The highest level of SC-Spm in AxA pollination model could affect the stiffening of pollen tube wall or of stylar cell wall, participating in the inhibition of tube growth. Moreover, the conjugation of PAs to HCAs, may represent a mechanism to counteract the cytotoxic effect due to HCAs (Kefeli et al., 2003).

The ^1^H NMR metabolomic analysis, which allowed comparing the metabolic patterns of the three classes, showed that pollination increased the overall metabolism, inducing a consumption of stored glucose, and increasing production of fatty acids, amino acids and organic acids, including also GABA and p-hydroxybenzoyc acid. The shikimate pathway was also enhanced, resulting in an increased production of phenolics, in particular flavonoids, confirmed by the analysis of total flavonoid content showing a significant increasing of these metabolites in AxA samples.

Moreover, both pollinated styles showed an increased concentration of alkaloid trigonelline. This compound is a nicotinamide metabolite involved in plant cell cycle regulation and oxidative stress (Perchat et al., 2018). Differences were also found between compatible (AxW) and incompatible (AxA). In particular, AxA showed generally higher metabolites content than AxW, except for sucrose, which was more concentrated in AxW. Signals due to a cynnamoil-derivative appeared also increased in AxA. This can reflect the huge accumulation of SC-Spm observed in SI pollinated styles.

Recent data from literature report that flavonols enhanced pollen development by acting as ROS scavengers and reducing the abundance of ROS that occurred when plant are exposed to stress (Muhlemann et al., 2018). The observation that maize and petunia mutants lacking chalcone synthase (CHS) activity were not only deficient in flavonoids, but were also male sterile suggested that flavonoids might be involved in pollen fertility (Mo et al., 1992; Muhlemann et al., 2018). These results reveal that flavonol metabolites regulate plant sexual reproduction under both normal and stress conditions (i.e., high temperatures) by maintaining ROS homeostasis. When pollen of CHS-deficient plants land on stigmas of wild-type plants, the mutant pollen is partially functional; this is because stigma release a diffusible factor that allows pollen to be functional at pollination. Moreover, this evidence led to the isolation and identification of kaempferol as a pollen germination-inducing constituent in wild-type petunia stigma extracts (Mo et al., 1992; Muhlemann et al., 2018).

Being flavonoids antioxidant molecules (Muhlemann et al., 2018), their increased level in AxA in respect to AxW, might represent a response to oxidative stress, which takes place during SI (Serrano et al., 2015). Although ROS are necessary for pollen tube growth, a misregulated ROS homeostasis, leading to exceeding accumulation of these radical species, causes damage to cell structures (Daher and Geitmann, 2011; Speranza et al., 2012; Sewelam et al., 2016). Increasing PAs, which were also found in AxA, might be related to this ROS imbalance. In fact, exogenous PAs alter ROS levels in *Pyrus pyrifolia* (Wu et al., 2010; Aloisi et al., 2015) and during the SI response (Wang et al., 2010; Jiang et al., 2014).

## Conclusion

The results of this study show that pollination process produces an intense metabolic activity, as emerged from the high accumulation of primary and secondary metabolites in pollinated styles compared to not pollinated ones. Moreover, a clear difference in the metabolomics profile exists between compatible-pollinated and SI-pollinated styles. In particular, during the self-pollinated response TGase activity increases, as well as the level of polyamines conjugated to hydroxycinnamic acids and other small molecules, and the shikimate pathways is induced, resulting in an increased production of phenolics, in particular flavonoids. These could be overproduced in the attempt to overcome the oxidative stress occurring during the SI response. On the other hands, the conjugation of hydroxycinnamic acids with aliphatic polyamines can moderate the reported cytotoxicity of free hydroxycinnamic acids. Taken together, the activation of TGase and the increase in conjugated and bound forms of polyamines can contribute to cause the rigidification of the cell wall and the decrease in its extensibility, thus impairing the pollen tube growth, which is the final event in the SI response.

## Data Availability

All datasets for this study are included in the manuscript and the Supplementary Files.

## Author Contributions

SD and FA designed the experiments. IA and SD produced the plant material. MM, FA, IA, GP, GC, LP, and CF performed the experiments, analyzed the data, and prepared the figures. MM, SD, FA, FP, and IA planned the research and interpreted the data. SD and MM wrote the manuscript with contributions from all other authors.

## Conflict of Interest Statement

The authors declare that the research was conducted in the absence of any commercial or financial relationships that could be construed as a potential conflict of interest.
