# Supplementary material for: Compatible and Incompatible Pollen-Styles Interaction in Pyrus communis L. Show Different Transglutaminase Features, Polyamine Pattern and Metabolomics Profiles
Source: Front Plant Sci. 2019 Jun 7;10:741. doi: 10.3389/fpls.2019.00741 (PMC6584118; doi:10.3389/fpls.2019.00741)
Supplement: Supplementary file 1 [file Table_1.DOCX]

**Supplementary Image Captions**

**Supplementary Image 1.** Styles of *Pyrus communis* L. cv Abbé Fétel pollinated on entire plant with compatible Williams pollen (AxW) stained with anilin blue (A, B, C). Several pollen grains (PG) adhering on the stigmatic papillae (P) are visible. Pollen tubes (arrows) penetrating along the style are visible because of the staining of their callosic cell wall. Bars: 100 μm.

**Supplementary Image 2.** Styles of *Pyrus communis* L. cv Abbé Fétel pollinated on entire plant with incompatible Abbé Fétel pollen (AxA) stained with anilin blue (A, B, C). Several pollen grains (PG) deposited on the surface of stigmatic papillae (P) are visible. Only a few pollen grains emitted a pollen tube (arrows) that are hardly observable along the style; in addition, the few pollen tubes appeared as bent and distorted. Bars: 100 μm.

**Supplementary Image 3.** NP styles of pear (A, B) on entire plant. Pollen grains are not visible as well as pollen tubes. The fluorescence signal was captured at higher exposure times to visualize the stylar cells. Bars: 100 μm.

**Supplementary Image 4**. (A) Compatible pollination (AxW) on pear styles on sprigs. It is possible to observe several pollen tubes that penetrate the stigma and grow along the style (arrows). Bar: 100 um. (B) Incompatible pollination (AxA) on pear styles on branches. In this case, growth of pollen tubes is strongly reduced and it usually stops in the stigma. Bar: 100 mm. (C) Example of unpollinated styles; no pollen tubes are visible. Bar: 100 mm.
