# Supplementary figures and images for: Compatible and Incompatible Pollen-Styles Interaction in Pyrus communis L. Show Different Transglutaminase Features, Polyamine Pattern and Metabolomics Profiles
Source: Front Plant Sci. 2019 Jun 7;10:741. doi: 10.3389/fpls.2019.00741 (PMC6584118; doi:10.3389/fpls.2019.00741)

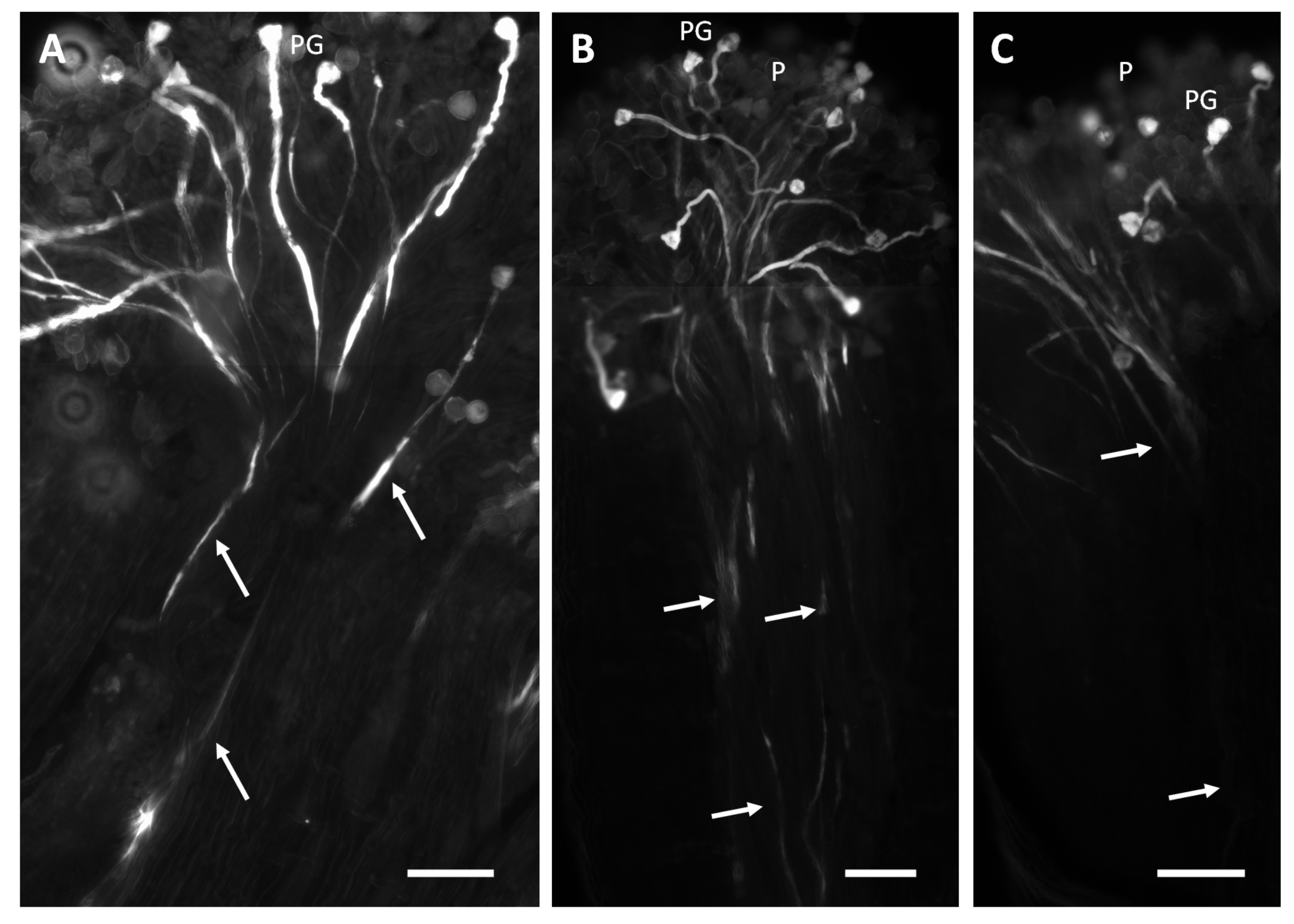

Supplement: Supplementary file 2 [file Image_1.JPEG]

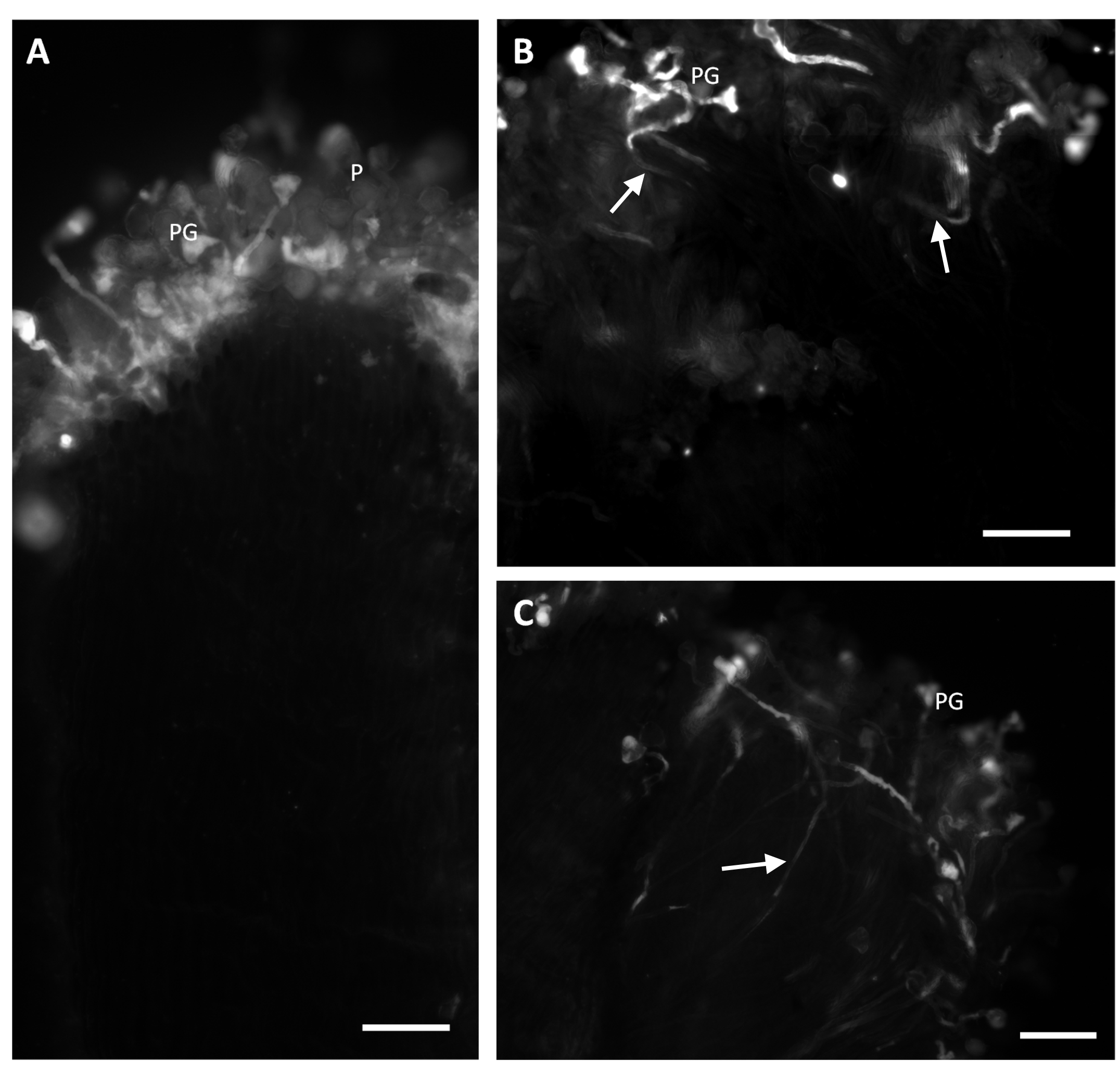

Supplement: Supplementary file 3 [file Image_2.JPEG]

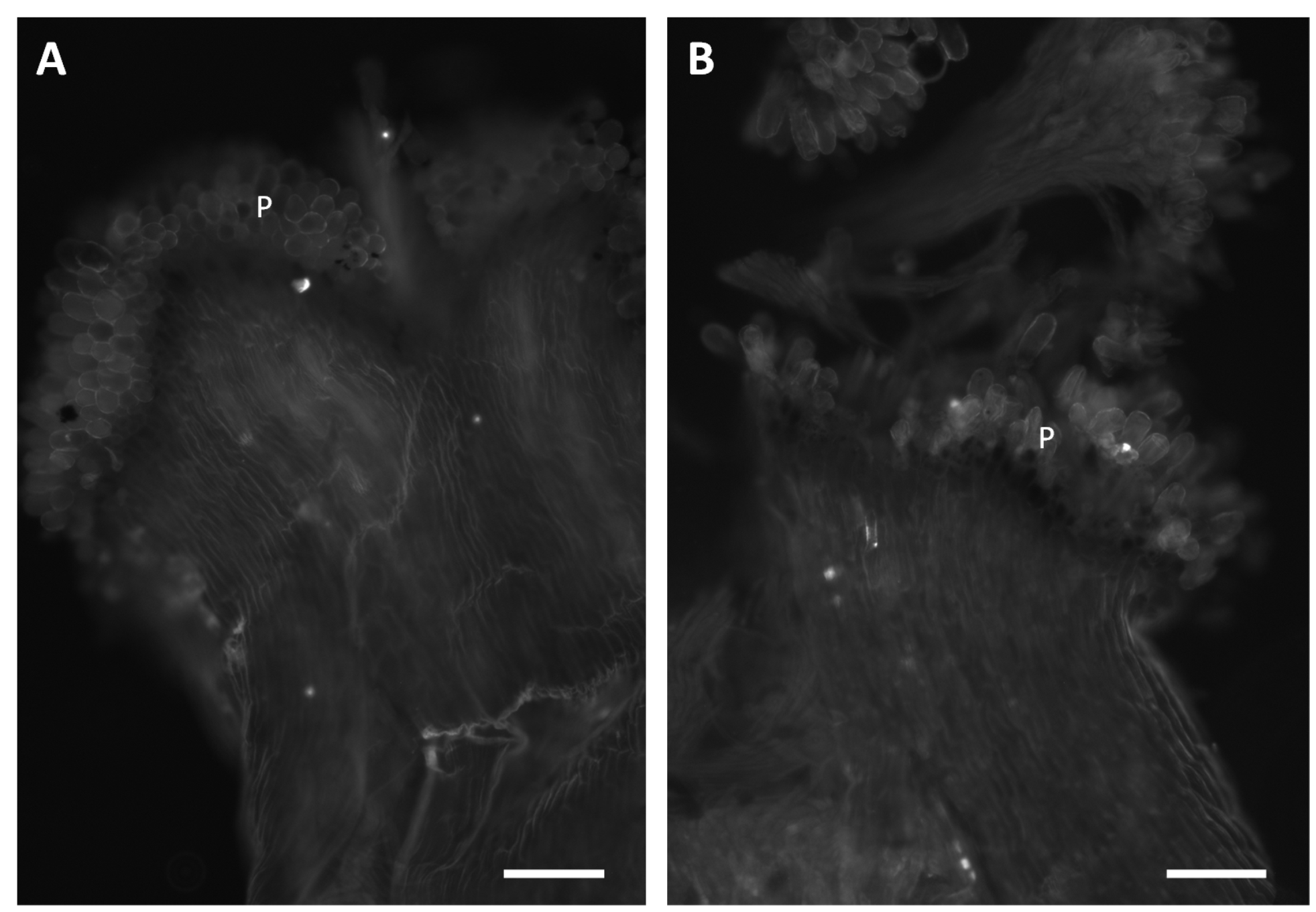

Supplement: Supplementary file 4 [file Image_3.JPEG]

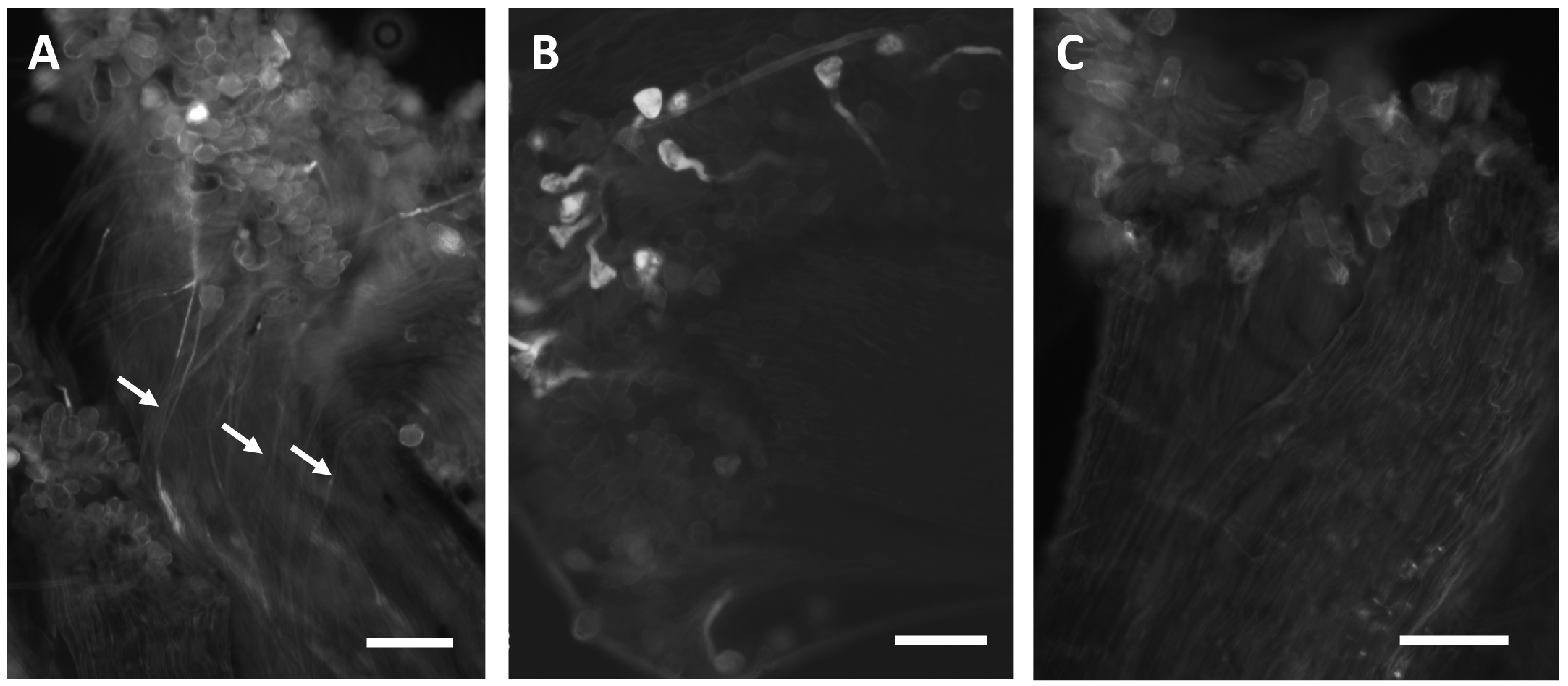

Supplement: Supplementary file 5 [file Image_4.JPEG]
